# Nonpaternity and Half-Siblingships as Objective Measures of Extramarital Sex: Mathematical Modeling and Simulations

**DOI:** 10.1155/2017/3564861

**Published:** 2017-08-22

**Authors:** Ryosuke Omori, Nico Nagelkerke, Laith J. Abu-Raddad

**Affiliations:** ^1^Division of Bioinformatics, Research Center for Zoonosis Control, Hokkaido University, Sapporo, Hokkaido, Japan; ^2^JST, PRESTO, 4-1-8 Honcho, Kawaguchi, Saitama 332-0012, Japan; ^3^Infectious Disease Epidemiology Group, Weill Cornell Medical College in Qatar, Cornell University, Qatar Foundation, Education City, Doha, Qatar; ^4^Department of Healthcare Policy and Research, Weill Cornell Medical College, Cornell University, New York, NY, USA; ^5^Institute of Public Health, College of Medicine and Health Science, United Arab Emirates University, Al Ain, UAE; ^6^Department of Public Health, Erasmus MC, University Medical Center Rotterdam, Rotterdam, Netherlands; ^7^Liverpool School of Tropical Medicine, Liverpool, UK

## Abstract

**Background:**

Understanding the epidemiology of HIV and other sexually transmitted infections (STIs) requires knowledge of sexual behavior, but self-reported behavior has limitations. We explored the reliability and validity of nonpaternity and half-siblings ratios as biomarkers of current and past extramarital sex.

**Methods:**

An individual-based Monte Carlo simulation model was constructed to describe partnering and conception in human populations with a focus on Sub-Saharan Africa (SSA). The model was parameterized with representative biological, behavioral, and demographic data.

**Results:**

Nonpaternity and half-siblings ratios were strongly correlated with extramarital sex, with Pearson correlation coefficients (PCC) of 0.79 (95% CI: 0.71–0.86) and 0.77 (0.68–0.84), respectively. Age-specific nonpaternity ratios correlated with past extramarital sex at time of conception for different scenarios: for example, PCC, after smoothing by moving averages, was 0.75 (0.52–0.89) in a scenario of steadily decreasing nonmarital sex and 0.39 (0.01–0.73) in a scenario of transient drops in nonmarital sex. Simulations assuming self-reported levels of extramarital sex from Kenya yielded nonpaternity levels lower than global nonpaternity data, suggesting sizable underreporting of extramarital sex.

**Conclusions:**

Nonpaternity and half-siblings ratios are useful objective measures of extramarital sex that avoid limitations in self-reported sexual behavior.

## 1. Background

Large generalized HIV epidemics have been observed only in Sub-Saharan Africa (SSA) [1]. While HIV acquisition globally is associated with specific high-risk sexual or injecting-drug behaviors, HIV prevalence in SSA has reached large levels even in the general population [2]. Several factors have been suggested to clarify the striking disparity in epidemic evolution between SSA and other regions, and most of these included explanations at the intersection of sexual behavior and host and virus biology [2, 3]. Despite progress, the factors underlying this fulminant transmission remain debated [3]. Understanding these factors has become further compelling by the large recent reductions in HIV prevalence observed across SSA and potential links to changes in sexual behavior [4].

HIV spreads in sexual networks that are connected. Connectivity and other network properties are determined by the collective sexual behavior of individuals, such as average of, and heterogeneity in, rates of partner change [5–7]. As self-reported numbers of sexual partners in SSA do not tend to differ from other parts of the world [8, 9], it has been proposed that higher concurrency, that is, having multiple partners simultaneously, rather than higher rates of partner change, explains observed HIV prevalence in SSA [10–12]. This has given rise to debates [7, 13–15], which in the context of insufficient reliable data are difficult to settle.

While extensive self-reported data on sexual behavior have become available in recent decades, the sensitive nature of sexual behavior and nonrandom social-desirability and recall biases, among other biases, raise questions about the accuracy and reliability of such data [17–24]. Studies have repeatedly demonstrated serious limitations to these data [25, 26], such as discrepancies in reporting between women and men [8].

To understand sexual behavior and its temporal trends and to capture behavioral determinants of sexually transmitted infections (STIs), reliable behavioral data are required. Representative proxy biomarkers would seem to offer a solution. Currently, however, there are few potential biomarkers. Established forensic techniques, such as counting Y-chromosomes in vaginal swabs, may provide a measure of recent unprotected sex with multiple partners, but obtaining such swabs is problematic for logistical and ethical concerns.

As an alternative, Abu-Raddad and Nagelkerke [30] proposed nonpaternity, established by DNA mismatches, as an objective measure/biomarker of sexual risk behavior including concurrency. Nonpaternity occurs when the biological father of a child differs from the putative father, often the husband or stable partner of the mother [16]. It would seem reasonable that nonpaternity is a reflection of extramarital sex at time of conception, which reflects partner concurrency. While no nonpaternity studies appear to have been conducted in SSA [30], studies in other regions suggest rates of around 2% in high paternity confidence unions [16]. Though representative data may not be available in SSA, some genetic studies involving trios of a child and its parents routinely check for nonpaternity. A study from Gambia, for example, reported an 8% prevalence of “false pedigrees” [31], highlighting data feasibility for this biomarker in SSA.

Against this background, we conducted in silico simulations of sex partnering and conception in SSA to provide proof-of-concept demonstration study for nonpaternity as an objective measure of extramarital sex. Our study addresses the following questions: (1) Can nonpaternity provide a statistically reliable and valid measure of extramarital sex partnering? (2) Can half-siblingships, a measure introduced here as a second measure of extramarital sex, provide also a statistically reliable and valid measure of extramarital sex partnering? (3) Can age-specific nonpaternity provide a statistically reliable and valid measure of decades-long secular trends in extramarital sex partnering, and can it capture historical changes in extramarital sex due, for example, to the onset of the HIV epidemic? We further investigated whether the range of global nonpaternity data is consistent (broadly) with self-reported levels of extramarital sex in SSA.

Of note here, although we use the term “extramarital sex” for conventional reasons, we use this term more broadly to encompass sex with a nonmain partner (for women who have a main partner). The distinction between “nonmain partner” and “extramarital sex” may depend on the specific society in which the nonpaternity and half-siblingships measures are used. In most societies, however, most women have one “regular,” “main,” or “marital” partner, one that is recognized by society.

## 2. Methods

### 2.1. Mathematical Model

We constructed an individual-based Monte Carlo simulation model describing the dynamics of sex partnering, conception, and birth and death in a given population. Each individual woman is born, dies, forms/dissolves marital/nonmarital partnerships, becomes pregnant, and gives birth of a new infant at event-specific probabilities at each time step in each simulation run. A population of 20,000 women is assumed (chosen for computational reasons), but this population varies in size with time by random birth and death events.

Marital partnerships are formed (and dissolved) for ages >15 years with absence of (female) polygamy. Nonmarital partnerships are formed (and dissolved) for ages >15 years among both unmarried (premarital sex) and married (extramarital sex) women, but at different rates. Conception is modeled factoring woman's age, type of sexual partnership (premarital, marital, and extramarital), age-specific probability of conception per coital act, age-specific frequency of coital acts, condom use, and distribution of sexual acts among married women (marital sex versus extramarital sex). No new conceptions can occur during the gestational period. The reduction in conception probability with condom use is modeled factoring condom efficacy in preventing conception and partnership-specific condom use coverage. Remarriage rates are assumed independent from nonmarital partnering rates.

Further details on model structure can be found in the Additional File 1 (see Supplementary Material available online at https://doi.org/10.1155/2017/3564861).

### 2.2. Data Sources

Rate of marriage formation was estimated using maximum likelihood applied to Kenya's 2008-2009 Demographic and Health Survey (DHS) data for the age-specific prevalence of current marriage (Additional File 1) [27]. Average marriage duration was set at 20 years as informed also by Kenya's 2008-2009 DHS data [27]. Figure S1 of Additional File 1 shows a comparison between model prediction and DHS data for the age-specific prevalence of current marriage.

Rates of premarital and extramarital partnership formation were estimated also using Kenya's 2008-2009 DHS data [27] per the methodology of Omori et al. [8]. For specific analyses (note Plan of Analysis), broader ranges of nonmarital sex rates were used as required by analysis, and these were based on nonmarital sex rates in SSA as derived for 20 countries by Omori et al. [8]. Age variation of nonmarital sex rates was calibrated based on analysis of United Kingdom National Survey of Sexual Attitudes and Lifestyle data [32]. Age-specific population size was estimated using Kenya's 2008-2009 DHS data [27] and projections of the Population Division of the United Nations Department of Economic and Social Affairs [33].

The (biological) per coital act age-specific probability of effective fecundability was set as estimated by Weinstein et al. based on United States data [34]. Age-specific frequency of coital acts was also set based on data reported by Weinstein et al. [34]. Condom efficacy in preventing conception was set as estimated by Davis and Weller [29]. Partnership-specific condom use coverage was set based on Kenya's 2008-2009 DHS data for condom use in last sex act [27]. Fraction of coital acts that are with the spouse, as opposed to an extramarital partner, among married women (*φ*_*m*_) was set at values varying over a wide range as required by analysis (note Plan of Analysis). Gestational period was assumed to follow a normal distribution with a mean and standard deviation based on data reported by Jukic et al. [28]. Age-specific death rate was set as reported by the Population Division of the United Nations Department of Economic and Social Affairs [33].

Parameter values used in our model are summarized in Table 1 and Table S1 of Additional File 1.

### 2.3. Epidemiologic Measures

We define nonpaternity events as children born from extramarital sex among married women. Accordingly, we define the* nonpaternity ratio* at time *t* (NPR(*t*)) as

(1) $$\begin{array}{c} \text{NPR}\left(\;t\;\right)=\frac{\left[\text{number of nonpaternity events among newly born infants over the last 12 months at time }t\right]}{\left[\text{number of newly born infants over the last 12 months at time }t\right]}. \end{array}$$

Analogously, we define a generalization of this ratio, the *x-age nonpaternity ratio* (NPR_*x*_(*t*)), to capture occurrence of nonpaternity in the past, that is, *x* years ago, based on a cross-sectional measure of nonpaternity in the present (i.e., at time *t*):

(2) $$\begin{array}{c} NPR_{x}\left(\;t\;\right)=\frac{\left[\text{number of nonpaternity events among those aged between }x\text{ and }x+1\text{ years and alive at time }t\right]}{\left[\text{number of all individuals aged between }x\text{ and }x+1\text{ years and alive at time }t\right]}. \end{array}$$

NPR_*x*_(*t*) is basically the* age-specific nonpaternity ratio* and NPR(*t* − *x*) = NPR_*x*_(*t*); that is, this measure provides the nonpaternity ratio *x* years back in time.

Since our aim is to assess whether nonpaternity can measure extramarital sex, we define the* extramarital partnership ratio* at time *t* (EPR(*t*)) as

(3) $$\begin{array}{c} EPR\left(\;t\;\right)=\frac{\left[\text{number of extramarital partnerships over the }l\text{ast 12 months among married women at time }t\right]}{\left[\text{number of all partnerships over the last 12 months among married women at time }t\right]}. \end{array}$$

We propose also a second proxy biomarker measure of extramarital sex, the ratio of half-siblings among children of the same mother. Specifically, we define the* half-siblings ratio* at time *t* (HSR(*t*)) as
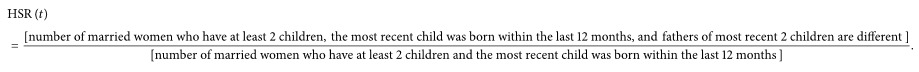
(4)


### 2.4. Plan of Analysis

#### 2.4.1. Nonpaternity as a Measure of Extramarital Sex Partnering

**Figure 1:**
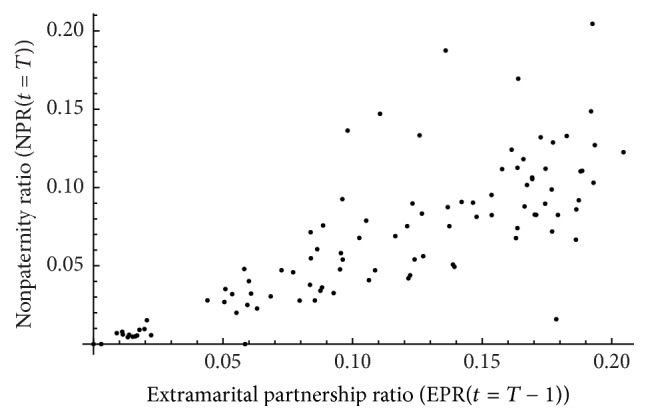
Nonpaternity as a measure of extramarital sex. Association between nonpaternity ratio (NPR(*t* = *T*)) and extramarital partnership ratio (EPR(*t* = *T* − 1)) across simulations assuming observed levels of self-reported extramarital sex partnering in Sub-Saharan Africa.

We assessed whether nonpaternity can provide a statistically reliable and valid measure of extramarital sex partnering within the context of SSA. This was addressed by assessing the correlation at some time *t* = *T* between NPR(*t* = *T*) and EPR(*t* = *T* − 1) across simulations (Figure 1). EPR(*t*) was assessed at *t* = *T* − 1, instead of *t* = *T*, to accommodate for the gestational period. Although the mean gestational period is less than a year (267 days [28]), the correlation was assessed at a one-year time shift for simplicity and computational tractability. In each simulation, means and variances of premarital and extramarital sex rates were drawn randomly from SSA ranges of means and variances [8, 27]. In absence of data, *φ*_*m*_ was set at 0.7 as a plausible value. Other values did not alter the results but affected the 95% confidence interval (CI) of the correlation. A total of 100 simulations were conducted and each simulation was run for 101 years with 50 years “burn-in,” thereby generating a set of 10,000 measures of NPR(*t* = *T*) and EPR(*t* = *T* − 1) across which the correlation was assessed. Pearson's correlation coefficient (PCC) was calculated with the 95% CI estimated by Fisher's* z*-transformation.

#### 2.4.2. Half-Siblingships as a Measure of Extramarital Sex Partnering

**Figure 2:**
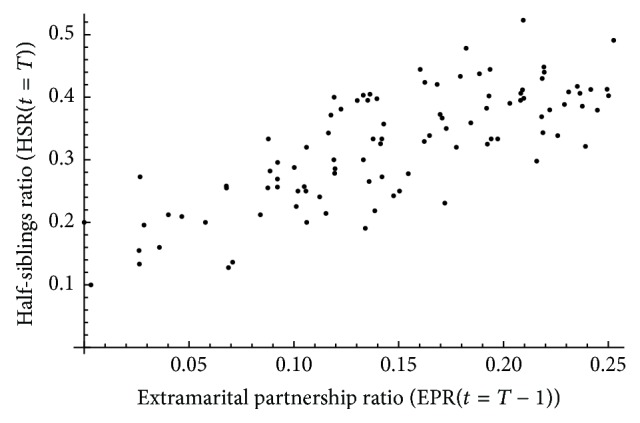
Half-siblingships as a measure of extramarital sex. Association between half-siblings ratio (HSR(*t* = *T*)) and extramarital partnership ratio (EPR(*t* = *T* − 1)) across simulations assuming observed levels of self-reported extramarital sex partnering in Sub-Saharan Africa.

We assessed whether half-siblingships can provide a statistically reliable and valid measure of extramarital sex partnering within the context of SSA. This was addressed by assessing the correlation at *t* = *T* between HSR(*t* = *T*) and EPR(*t* = *T* − 1) across simulations (Figure 2). EPR(*t*) was assessed at *t* = *T* − 1 to accommodate for the gestational period. In each simulation, means and variances of premarital and extramarital sex rates were drawn randomly from SSA ranges of means and variances [8, 27]. *φ*_*m*_ was set at 0.7. A total of 100 simulations were conducted and each simulation was run for 101 years with 50 years “burn-in,” thereby generating a set of 10,000 measures of HSR(*t* = *T*) and EPR(*t* = *T* − 1) across which the correlation was assessed. PCC was calculated with the 95% CI estimated by Fisher's* z*-transformation.

#### 2.4.3. Nonpaternity as a Measure of Historical Variation in Extramarital Sex Partnering

**Figure 3:**
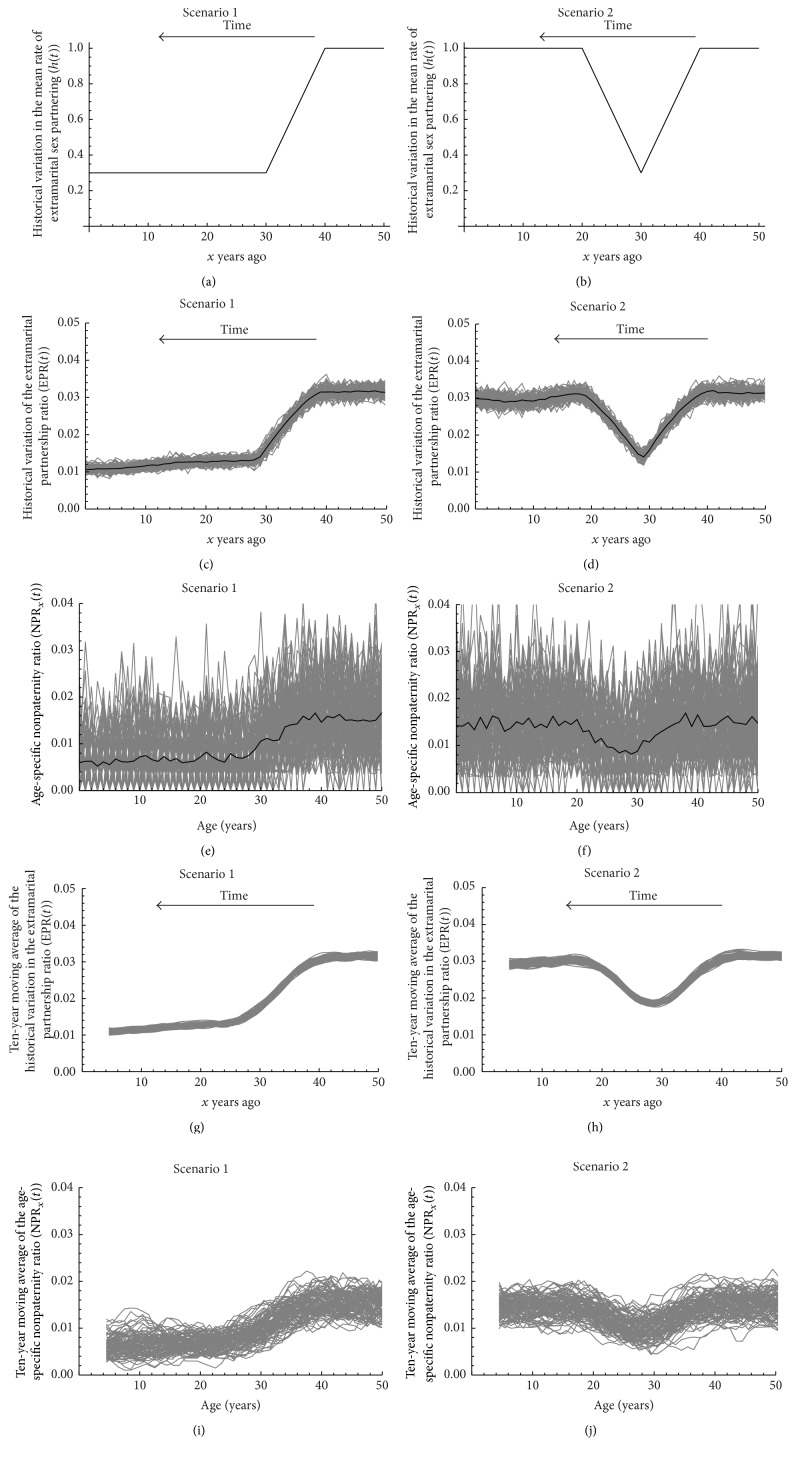
Nonpaternity as a measure of historical variation in extramarital sex. ((a) and (b)) Two different scenarios for the historical variation in the rate of extramarital sex partnering. ((c) and (d)) Historical variation of the extramarital partnership ratio (EPR(*t*)) in both scenarios. ((e) and (f)) The age-specific nonpaternity ratio (NPR_*x*_(*t*)) in both scenarios. ((g) and (h)) Ten-year moving average of the historical variation in the extramarital partnership ratio (EPR(*t*)) in both scenarios. ((i) and (j)) Ten-year moving average of the age-specific nonpaternity ratio (NPR_*x*_(*t*)) in both scenarios. Gray lines in panels (c)–(j) show sample paths of each simulation run. Black lines in panels (c)–(f) show the means over the 100 simulation runs.

We assessed whether age-specific nonpaternity can provide a statistically reliable and valid measure of historical trends in extramarital sex partnering within the context of SSA. To this end, we varied the nonmarital sex rates with a time-dependent factor *h*(*t*). We explored two scenarios for *h*(*t*) (Figure 3). First, nonmarital sex decreased steadily for 10 years and then plateaued (Figure 3(a)). This scenario is motivated by apparent changes in sexual risk behavior in SSA following the emergence of the HIV epidemic [4]. Second, nonmarital sex had only a transient decrease (Figure 3(b)). The lowest nonmarital sex level in both scenarios was set by Kenya's 2008-2009 DHS data [8, 27]. The latter is at 30% of the peak level (Figures 3(a) and 3(b)), as informed by estimates of the historical reduction in sexual risk behavior in SSA following the emergence of the HIV epidemic [4]. *φ*_*m*_was set at 0.7.

To address the research question, we assessed the correlation at *t* = *T* between NPR_*x*_(*t* = *T*) and EPR(*t* = *T* − *x* − 1) across simulations. A total of 100 simulations were conducted and each simulation was run for 201 years to calculate 100 measures of the age-specific nonpaternity ratio for those aged 0–50 years (i.e., NPR_*x*_(*t*) for 0 ≤ *x* ≤ 50, 1 ≤ *t* ≤ 100, and “burn-in” for 50 years). Accordingly, a set of 10,000 measures of NPR_*x*_(*t* = *T*) and EPR(*t* = *T* − *x* − 1) were generated across which the correlation was assessed. PCC was calculated with the 95% CI estimated by Fisher's* z*-transformation.

#### 2.4.4. Consistency between Nonpaternity Data and Self-Reported Extramarital Sex in SSA

**Figure 4:**
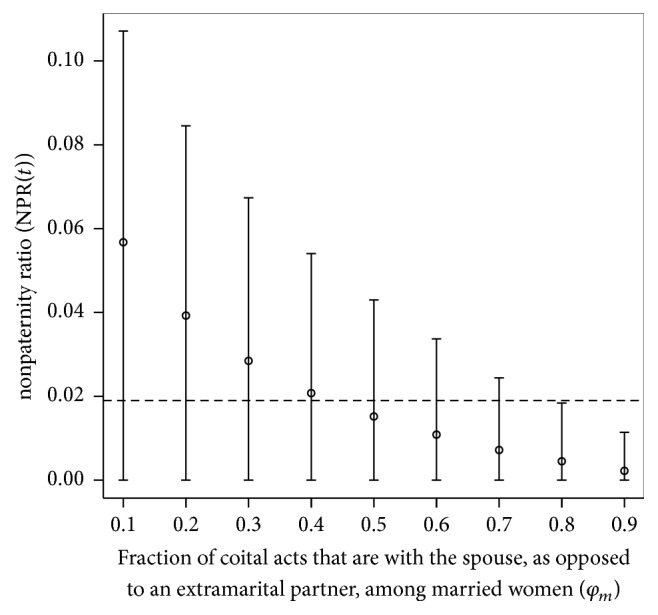
Consistency between global nonpaternity data and self-reported extramarital sex in Sub-Saharan Africa. Mean and 95% confidence interval of the nonpaternity ratio (NPR(*t*)) over 100 simulations at different values of the fraction of coital acts that are with the spouse, as opposed to an extramarital partner, among married women. The dashed black line shows a typical intermediate value for the nonpaternity ratio per a global review of nonpaternity data [16].

We explored the consistency between global nonpaternity data and self-reported extramarital sex among women in SSA, to determine whether self-reported data could be underestimating actual levels. This was done by conducting simulations at variable values of *φ*_*m*_, in the range of 0.1–0.9 and a step of 0.1, while fixing nonmarital sex rates per Kenya's 2008-2009 DHS data [8, 27]. A total of 100 simulations were conducted for each value of *φ*_*m*_, and mean NPR(*t* = *T*) and 95% CI were calculated across these simulations. The set of means was then compared to nonpaternity data provided through a global review (Figure 4) [16].

## 3. Results

Figure 1 shows the association between nonpaternity ratio (NPR(*t* = *T*)) and extramarital partnership ratio (EPR(*t* = *T* − 1)) across the generated simulations. NPR(*t* = *T*) was strongly correlated with EPR(*t* = *T* − 1), with a PCC of 0.79 (95% CI: 0.71–0.86).

Figure 2 shows the association between half-siblings ratio (HSR(*t* = *T*)) and EPR(*t* = *T* − 1) across the generated simulations. HSR(*t* = *T*) was strongly correlated with EPR(*t* = *T* − 1), with a PCC of 0.77 (95% CI: 0.68–0.84).

The age-specific nonpaternity ratio, as measured among currently living cohorts, correlated with past extramarital sex behavior of the mothers at the time of conception of each age cohort, as seen by comparing Figures 3(e)–3(c) and 3(f)–3(d). PCC was 0.29 (95% CI: 0.15–0.39) for the scenario of decreasing nonmarital sex (Figure 3(a)) and 0.10 (95% CI: −0.04–0.27) for the scenario of only transient decrease in nonmarital sex (Figure 3(b)). The association was more visually clear by using moving averages to smoothen the stochastic fluctuations in the processes of extramarital sex partnering and conception (Figure 3(i) versus Figure 3(g) and Figure 3(j) versus Figure 3(h)). After filtering out random “noise,” the PCC was 0.75 (95% CI: 0.52–0.89) for the scenario of decreasing nonmarital sex and 0.39 (95% CI: 0.01–0.73) for the scenario of only transient decrease in nonmarital sex.

Figure 4 shows the intermediate value for the empirical nonpaternity ratio per a global review of data [16], compared with the simulated nonpaternity ratio at different values of *φ*_*m*_ assuming extramarital sex rates per Kenya's 2008-2009 DHS data [8, 27]. The mean simulated nonpaternity ratio was lower than the intermediate global line for *φ*_*m*_ ≥ 0.5, and the 95% CI did not overlap with the line for *φ*_*m*_ ≥ 0.8. Large values for *φ*_*m*_ were not consistent with empirical data, implying either that as much as half of coital acts could be with extramarital partners or that self-reported extramarital sex partnering underestimates actual levels, or some combination of these two factors (coital acts frequency within and outside marital partnerships versus number of extramarital partners among married women).

## 4. Discussion

We presented an approach to study various public health research questions relating to reproduction, fertility, sexual behavior, and STIs. The approach was implemented in a study of nonpaternity and whether nonpaternity can provide an objective proxy biomarker of extramarital sex and concurrency of sexual partnerships. We found that both nonpaternity and half-siblings ratios provide statistically reliable and valid measures of extramarital sex, thereby avoiding key limitations in self-reported sexual data. We also found that the age-specific nonpaternity ratio, as measured among currently living cohorts, can provide a measure of decades-old trends in past extramarital sex, such as around the onset of the HIV epidemic in SSA. We further found that self-reported extramarital sex data are likely to underestimate actual extramarital sex. These findings have important implications for measuring and understanding the risk and dynamics of STIs including HIV and provide insights about reproductive health as well as female sexuality, a subject of conflicting evidence [35].

Our study highlights the opportunity of using nonpaternity as an objective measure of extramarital sex and adds to the evidence indicating that self-reported sexual data are prone to serious biases that limit their utility in understanding STI dynamics [8, 17–22, 24–26]. While our study provided the proof of concept for the utility of nonpaternity, concrete results of extramarital sex in SSA can only be attained when empirical nonpaternity data are analyzed using the theoretical approach presented here. Hardly any nonpaternity data, however, has been collected in SSA [16, 36], but it is feasible for such data to be collected in anonymous and nonstigmatizing contexts using existing or future genetic studies, such as those involving trios of a child and its parents that routinely check for nonpaternity [31]. These studies may also avoid potential selection bias in nonanonymized studies and could be conducted as secondary data analyses on existing samples. There could be also an opportunity for such studies to be carried out using probability-based samples by nesting them in national surveys involving anonymous blood specimen collection such as DHS.

In addition to nonpaternity ratio, we introduced half-siblings ratio, which captures simultaneously two types of partnerships among married women, extramarital and remarriage. Therefore, this measure is of interest in its own right as a measure of sexual and STI risk. It would seem reasonable to assume that a mother of five children, all from the same father, had lower sexual risk behavior over her reproductive period, than a mother whose five children all had different fathers, regardless of whether these fathers were marital or extramarital partners. While nonpaternity data requires the availability of the putative father, thereby potentially introducing selection bias, this measure depends only on mother and children data.

Our study has several limitations. Although the gestational period lasts for less than a year, we assessed the correlations at a one-year shift, but this only underestimates the correlations further supporting our hypothesis. While the age-specific nonpaternity measures past extramarital sex trends, the correlations were weaker for rapid changes highlighting the challenge of capturing transient variation using this measure (Figure 3). While nonpaternity measures collectively extramarital sex, it cannot distinguish between multiplicity of extramarital partners and multiplicity of coital acts with one or more extramarital partners.

Nonpaternity and half-siblingships are useful measures of extramarital sex, but these measures could be affected by several factors. Pregnancy is not necessarily a sensitive measure of exposure to condomless sex. Most sexual exposures will not lead to pregnancy and pregnancy risk can be influenced by other factors such as contraception use and fecundity. These factors can also change overtime, further complicating and potentially biasing the use of nonpaternity and half-siblingships as measures of extramarital sex.

Having said so, these factors may not affect the validity of nonpaternity and half-siblingships as measures if these factors act nondifferentially. For example, as long as hormonal contraceptive use is nondifferential with respect to having extramarital sex, it should not diminish the value of nonpaternity and half-siblingships as measures of extramarital sex on a population level. Moreover, nonpaternity and half-siblingships are not meant to be the definitive measures of extramarital sex. We advocate here for their use along with other objective measures of sexual risk behavior in a population, for example, the use of the prevalence of STIs to inform our knowledge of sexual risk behavior [7]. A complete picture of sexual risk behavior in a population may not be attainable without the concurrent use of several such independent and complementary measures.

Our study provided a proof of concept, but there are challenges in collecting empirical data. Identification of nonpaternity requires both genetic paternity and paternity confidence, but paternity confidence can be ambiguous. Nonpaternity could be due to covert adoptions, misidentified stepchildren, or sperm donations [16, 36]. Differentials in induced abortion can also bias estimates, although the impact is small if abortion rates are low [37]. There are also ethical challenges in nonpaternity studies, but these are manageable and should not hinder progress in empirical data.

Nonpaternity ratio can be defined in different ways, factoring often the degree of paternity confidence. We defined it as the fraction of children born from extramarital sex among married women, with no restriction on the perceived degree of paternity confidence. For nonpaternity to be a useful measure, it is critical to have a standardized definition. We propose our definition as a natural and practical definition that minimizes bias with paternity confidence perception.

## 5. Conclusions

Nonpaternity and half-siblings ratios are useful measures of extramarital sex, and age-specific nonpaternity provides a window into the past by assessing decades-old extramarital sex. Self-reported sexual data appear to underestimate extramarital sex, but nonpaternity can provide a trove of objective data to inform our understanding of STI dynamics and human sexuality. These data can also facilitate improved knowledge of HIV epidemics and their evolution and can inform concept and design of interventions, thereby optimizing impact of programs by better targeting of the drivers of infection transmission.

## Supplementary Material

Further details on the mathematical model and its data input parametrization.

## Figures and Tables

**Table 1:** Key model assumptions in terms of parameter values.

Description	Symbol	Value	Reference
Marriage rate	*α*	0.12 per year	Estimated from Kenya's 2008-2009 DHS data [27]

Mean duration of martial partnership	1/*μ*_marital_	20 years	Representative value informed by Kenya's 2008-2009 DHS data [27]

Mean duration of nonmarital (casual) sexual partnership	1/*μ*_casual_	6 months	Representative value

Gestation period			[28]
Mean		267 days	
Standard deviation		10 days	

Number of premarital sex partnerships among unmarried women over the last year			Estimated from Kenya's 2008-2009 DHS data [27]. Range is estimated based on DHS data for 20 Sub-Saharan African countries [8]
Mean		0.32 and a range of 0.0–1.0	
Variance		0.0087 and a range of 0.0–2.0	

Number of extramarital sex partnerships among married women over the last year			Estimated from Kenya's 2008-2009 DHS data [27]. Range is estimated based on DHS data for 20 Sub-Saharan African countries [8]
Mean		0.0088 and a range of 0.0–0.5	
Variance		0.0 with a range of 0.0–1.0	

Fraction of coital acts that are with the spouse as opposed to an extramarital partner	*φ*_*m*_	0.7 and a range of 0.1–0.9	Representative but variable by objective of each specific analysis

Partnership-specific condom use coverage	*C*_*v*_		Estimated from Kenya's 2008-2009 DHS data [27]
Unmarried women		0.23	
Marital sex among married women		0.019	
Extramarital sex among married women		0.074	

Efficacy of condoms in preventing conception	Eff	0.90	[29]

DHS: Demographic and Health Survey.

## Abbreviations

CI: Confidence interval
DHS: Demographic and Health Survey
DNA: Deoxyribonucleic acid
EPR: Extramarital partnership ratio
HIV: Human immunodeficiency virus
HSR: Half-siblings ratio
NPR: Nonpaternity ratio
PCC: Pearson correlation coefficient
SSA: Sub-Saharan Africa
STI: Sexually transmitted infection.
