# Supplementary material for: Nonpaternity and Half-Siblingships as Objective Measures of Extramarital Sex: Mathematical Modeling and Simulations
Source: Biomed Res Int. 2017 Aug 22;2017:3564861. doi: 10.1155/2017/3564861 (PMC5585552; doi:10.1155/2017/3564861)
Supplement: Supplementary file 1 — Further details on the mathematical model and its data input parametrization. [file 3564861.f1.docx]

**Supplemental Information**

**Non-paternity and half-siblingships as objective measures of extramarital sex: Mathematical modeling and simulations**

Ryosuke Omori^a,b,c,d*^, Nico Nagelkerke,^e,f,g^ and Laith J. Abu-Raddad^c,d^

^a^*Division of Bioinformatics, Research Center for Zoonosis Control, Hokkaido University, Sapporo, Hokkaido, Japan*

^b^*JST, PRESTO, 4-1-8 Honcho, Kawaguchi, Saitama, 332-0012, Japan*

^c^*Infectious Disease Epidemiology Group, Weill Cornell Medical College in Qatar, Cornell University, Qatar Foundation - Education City, Doha, Qatar*

^d^*Department of Healthcare Policy and Research, Weill Cornell Medical College, Cornell University, New York, New York, USA*

^e^*Institute of Public Health, College of Medicine and Health Science, United Arab Emirates University, Al Ain, United Arab Emirates*

^f^*Department of Public Health, Erasmus MC, University Medical Center Rotterdam, Rotterdam, Netherlands*

^g^*Liverpool School of Tropical Medicine, Liverpool, UK*

*Reprints or correspondence: Ryosuke Omori, PhD, Division of Bioinformatics, Research Center for Zoonosis Control, Hokkaido University, Sapporo, 001-0020, Japan. E-mail: omori@czc.hokudai.ac.jp

**Mathematical model**

We constructed an individual-based Monte Carlo simulation model describing the formation/dissolution of marital/non-marital (casual) partnerships and births and deaths. Each individual is born, dies, forms, and dissolves marital and non-marital sex partnerships with given rates or probabilities. Definition of each rate or probability can be found in the sections below. Population size at the beginning of each simulation run was set at 20,000. Parameter values used in our simulations are summarized in Table 1 of main text and Table S1.

*1. Formation and dissolution of marital partnerships*

Marital partnerships were formed and dissolved with annual rates of $\alpha$ and $\mu_{marital}$, respectively. We assumed that $\alpha$ and $\mu_{marital}$ to be age independent (for ages > 15 years, otherwise $\alpha=\mu_{marital}=0$) and identical for all women, with absence of (female) polygamy. To estimate the formation rate $\alpha$, the following mathematical model was employed for the dynamics of marital partnership formation and dissolution:

$\frac{dm(y)}{dy}=\alpha(1-m(y))-\mu_{marital}m(y)$ (1)

where $m(y)$ is the fraction of married people at age $y$*.*

The solution of this equation with the initial condition $m(15)=0$ is:

$m(y)=\frac{\alpha-\alpha\exp[-(a+\mu_{marital})(y-15)]}{\alpha+\mu_{marital}}$ (2)

Here, $1/\mu_{marital}$ is the average duration of marital partnership set at 20 years as informed by Kenya’s 2008-2009 Demographic and Health Survey (DHS) data [1]. $\alpha$ was estimated by fitting Equation 2 using maximum likelihood to Kenya’s 2008-2009 DHS data for the age-specific prevalence of current marriage [1]. Specifically, the probability distribution at age $y$ of the observed number of married women $k$ among those at age $y$ in a sample of size $n$ is $Bin\left(k;n,m\left(y\right)\right)$. This distribution, and Equation 2, defines the likelihood. Figure S1 shows a comparison between model estimate and DHS data for the age-specific prevalence of current marriage using our estimate $\alpha=0.12$.

*2. Formation and dissolution of non-marital (casual) partnerships*

Casual partnerships for the $x$-th woman with age $y$ and marital status $z$ (unmarried or married) were formed and dissolved with annual rates $\pi_{x,y,z}$ and $\mu_{casual}$, respectively. We assumed a mean non-marital (casual) partnership duration of six months ($1/\mu_{casual}$), constant over time and the same for all individuals.

Formation rates $\pi_{x,y,z}$ were assumed to factorize into an individual’s propensity to form extra-marital relationships and an age-specific profile as

$\pi_{x,y,z}=\psi_{x,z}\hat{\sigma}_{y}$ (3)

where $\psi_{x,z}$ is the mean acquisition rate of non-marital partnerships for the $x$-th woman with marriage status $z$ over her sexual life span. $\psi_{x,z}$ was estimated from DHS sexual behavior data [1] per the methodology of Omori *et al.* [2].

The parameter $\hat{\sigma}_{y}$ is the relative age-specific sexual partnership acquisition rate, relative to the mean sexual partnership acquisition rate averaged over the population of all ages:

$\hat{\sigma}_{y}=\left(\frac{\sum_{w} N_{w}}{\sum_{w} \sigma_{w}N_{w}}\right)\sigma_{y}$ (5)

Here $\sigma_{y}$ is the mean sexual partnership acquisition rate for age $y$, and $N_{w}$ is the age specific population size for age group $w$.

$\sigma_{y}$ was derived using the data of the United Kingdom National Survey of Sexual Attitudes and Lifestyle (NATSAL) per the analysis of Choi *et al.* [3]. $N_{w}$ was estimated using Kenya’s 2008-2009 DHS data [1].

*3. Conception*

Conception for women with age $y$ and partnership type $v$ occurs at a rate $\lambda_{y,v}$. Partnership type $v$ includes the categories of premarital partnerships of unmarried women, marital partnership of married women, and extramarital partnerships of married women.

$\lambda_{y,v}$ depends on the following factors: 1) age-specific probability of conception per coital act, 2) age-specific frequency of coital acts, 3) condom use, and 4) distribution of sexual acts among married women (marital sex versus extramarital sex). This dependence is captured by the equation:

(7)

Here, $\theta_{y}$ is the per-coital-act age-specific probability of effective fecundability for women with age $y$, as estimated by Weinstein *et al.* [4]. $\xi_{y}$ is the frequency of coital acts at age $y$, based on empirical data reported by Weinstein *et al.* [4].

$\varepsilon_{condom,v}$ is the reduction in the probability of conception due to condom use for partnership of type $v$. $\varepsilon_{condom,v}$ is calculated by the expression:

$\varepsilon_{condom,v}=\left(1-Eff\right)C_{v}+1-C_{v}$ (8)

Here $Eff$ is the efficacy of condoms in preventing conception, set at 0.90 per Davis and Weller [5]. $C_{v}$ is the partnership-specific condom use coverage, estimated by self-reported use of condoms in last sex act per Kenya’s 2008-2009 DHS data [1]. Accordingly, $C_{v}$ was estimated at 0.23 for premarital sex among unmarried women, 0.019 for marital sex among married women, and 0.074 for extramarital sex among married women.

describes the fraction of coital acts that occur in partnership of type $v$. is equal to 1 for unmarried women (all acts are casual sex acts as all partnerships are premarital partnerships). Meanwhile, for the marital partnership among married women, where is the fraction of coital acts that are with the spouse. Accordingly, for the extramarital partnerships among married women.

Lastly, $k$ is an overall constant used to adjust overall fertility, and consequently population growth and size.

The probability $\varpi_{y,v}$ of at least one conception during a specific period $T$ is then given by

$\varpi_{y,v}=1-\text{exp}\left[-\lambda_{y,v}T\right]$ (6)

*4. Birth and death*

After conception, no new conceptions can occur during the gestational period. The gestational period was assumed to follow a normal distribution with a mean of 267 days and a standard deviation of 10 days, based on empirical data as reported by Jukic *et al.* [6]. The age-specific death rate ($\delta_{y}$) was set using Kenya’s demographic indicators, as reported by the Population Division of the United Nations Department of Economic and Social Affairs [7].

**Figure S1.** **Marriage among women by age.** Fraction of married women out of all women as estimated by our model (solid line) compared with Kenya’s 2008-2009 DHS data (black dots) [1].

**
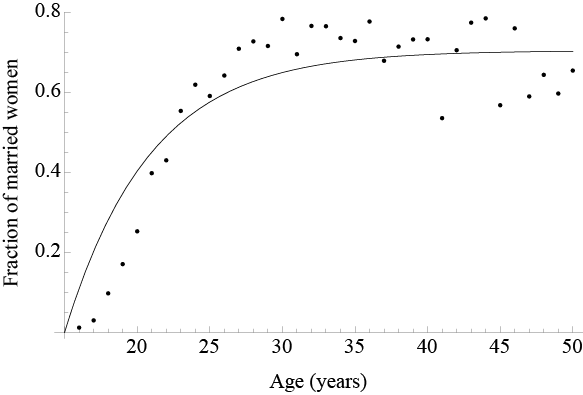
**

**Table S1.** Age-specific parameter values for the frequency of coital acts ($\xi_{y}$), mean sexual partnership acquisition rate ($\sigma_{y}$), and probability of effective fecundability ($\theta_{y}$).

| Age (years) | Frequency of coital acts per week ($\xi_{y}$) [4] | Mean sexual partnership acquisition rate per year ($\sigma_{y}$) [3] | Probability of effective fecundability per coital act ($\theta_{y}$) [4] |
| --- | --- | --- | --- |
| 0-14 | 0.00 | 0.000 | 0.000 |
| 15-19 | 3.90 | 0.850 | 0.028 |
| 20-24 | 3.50 | 1.200 | 0.050 |
| 25-29 | 3.20 | 0.610 | 0.056 |
| 30-34 | 2.90 | 0.330 | 0.054 |
| 35-39 | 2.50 | 0.250 | 0.050 |
| 40-45 | 2.20 | 0.190 | 0.044 |
| 45-49 | 1.90 | 0.140 | 0.025 |
| 50-54 | 1.50 | 0.095 | 0.006 |
| 55-59 | 1.20 | 0.065 | 0.000 |
| 60-64 | 0.87 | 0.045 | 0.000 |
| 65- | 0.00 | 0.000 | 0.000 |

**References**

1. MEASURE DHS, "Demographic and health surveys," Ed., ICF Macro, Calverton.

2. R. Omori, H. Chemaitelly and L. J. Abu-Raddad, "Dynamics of non-cohabiting sex partnering in sub-Saharan Africa: a modelling study with implications for HIV transmission," *Sex Transm Infect*, vol. 91, no. 6, pp. 451-457, 2015.

3. Y. H. Choi, M. Jit, N. Gay, A. Cox, G. P. Garnett and W. J. Edmunds, "Transmission dynamic modelling of the impact of human papillomavirus vaccination in the United Kingdom," *Vaccine*, vol. 28, no. 24, pp. 4091-4102, 2010.

4. M. Weinstein, J. W. Wood, M. A. Stoto and D. D. Greenfield, "Components of age-specific fecundability," *Population Studies*, vol. 44, no. 3, pp. 447-467, 1990.

5. K. R. Davis and S. C. Weller, "The effectiveness of condoms in reducing heterosexual transmission of HIV," *Fam Plann Perspect*, vol. 31, no. 6, pp. 272-279, 1999.

6. A. M. Jukic, D. D. Baird, C. R. Weinberg, D. R. McConnaughey and A. J. Wilcox, "Length of human pregnancy and contributors to its natural variation," *Human Reproduction*, vol. 28, no. 10, pp. 2848-2855, 2013.

7. United Nations Department of Economic and Social Affairs, Population Division and Population Estimates and Projections Section, "World population prospects, the 2012 revision," Ed., 2012. available: <http://esa.un.org/wpp/Excel-Data/population.htm>
